# Total knee arthroplasty following intramedullary tibial nailing

**DOI:** 10.1186/s43019-020-00055-2

**Published:** 2020-07-21

**Authors:** Evan J. Smith, Marilyn Heng, Hany S. Bedair, Christopher M. Melnic

**Affiliations:** Department of Orthopaedic Surgery, Massachusetts General Hospital/Newton Wellesley Hospital, Harvard Medical School, 55 Fruit Street, YAW 3700 – Suite 3B, Boston, MA 02114 USA

**Keywords:** Knee, Fracture, Arthroplasty, Tibial nail, Intramedullary nail

## Abstract

**Introduction/purpose:**

Total knee arthroplasty (TKA) in the setting of previous periarticular hardware increases resource utilization, readmissions, complications, and revision rates. Despite the frequency of intramedullary nail (IMN) fixation for tibial fractures, little guidance exists on the management of these patients and no series have reported on outcomes of patients undergoing TKA in the setting of a retained or removed IMN.

**Methods:**

This is a retrospective case series of patients who underwent TKA after IMN fixation of tibial fractures. Patient and case data, including need for hardware removal, staged vs non-staged procedures, operative time, and need for revision implants, were recorded. Postoperative data, including complications and revision, were recorded. Oxford Knee Score (OKS) was performed at follow-up.

**Results:**

Nine patients were identified consisting of eight women and one man. Follow-up ranged from 0.8–13 years. Non-staged removal of the intramedullary hardware occurred in three cases that had increased operative lengths recorded. There were no complications related to wound healing or infection. No patients required revision. Two of the three patients who underwent non-staged TKA developed arthrofibrosis requiring manipulation. OKS scores in patients who underwent non-staged surgery were consistently low.

**Conclusions:**

Conversion TKA after tibial IMN fixation can result in satisfying outcomes in many patients. However, intramedullary hardware presents challenges to TKA similar to more extensively studied conversion TKA scenarios. Removing hardware in either a staged or non-staged fashion results in increased resource utilization and imparts perioperative challenges with only theoretical benefits of one approach compared to the other. Increased stiffness may be associated with a non-staged approach to hardware removal and TKA. Several technical factors may permit component positioning without removal of hardware. Despite limitations, this is the first series to discuss this challenging clinical scenario and provides surgeons with technical guidance and data on operative outcomes.

## Introduction/purpose

Intramedullary nail (IMN) fixation has become the treatment of choice for most diaphyseal tibial fractures. Techniques for placing intramedullary nails (IMNs) in the tibia often require violation of the knee joint and may complicate future knee arthroplasty due to the position of the nail which challenges placement of the tibial components [1, 2].

Total knee arthroplasty (TKA) in the setting of previous periarticular hardware increases resource utilization, readmissions, mechanical complications, infectious complications, and overall revision rates [3–10]. Further, a history of fracture has been associated with increased risk of postoperative complications and infection when compared to cases involving hardware from non-fracture surgery [4, 5].

Previous series on the effect of periarticular hardware on TKA outcomes have focused on patients with distal femoral and proximal tibial plates for fracture or corrective osteotomy, patellar fixation, retained hardware from ligament reconstruction and tibial tubercle osteotomies [3, 8, 10–14]. Despite the frequency of IMN fixation for tibial fractures and the proximity to the knee joint, no series have specifically reported on outcomes of patients undergoing TKA in the setting of a retained or removed IMNs. Additionally, there is limited discussion on the optimal approach to conversion TKA in the setting of prior tibial IMN fixation. In the present study, we hypothesize that cases which utilized a staged approach to nail removal would result in increased wound and mechanical complications compared to cases utilizing a concurrent approach to nail removal.

## Methods

This is a retrospective case series of patients who underwent TKA after intramedullary nailing of tibial-shaft fractures. After obtaining Institutional Review Board (IRB) approval, our insitutional database was queried to identify patients who underwent both TKA and either removal of hardware or insertion of an intramedullary tibial nail. Cases were then manually reviewed and nine cases were identified of TKA after previous tibial IMN. Basic demographic data and case data were recorded. Case data included hardware removal or retention, staged vs non-staged hardware removal, operative time, and need for constrained or revision implants. Postoperative data including complications and revision were recorded for each patient. Complications included, wound breakdown, mechanical complications (arthrofibrosis, instability, loosening), and infection (superficial and deep). All patients underwent a telephone interview at the conclusion of the study to confirm that no additional complications or revisions had been performed since their last office visit. An Oxford Knee Score (OKS) was performed for each patient. Two patients were unable to be contacted. Due to the small patient numbers, statistical analysis did not meet adequate power to detect differences in complications or outcomes.

## Results

We identified nine patients who underwent TKA after having undergone a previous tibial IMN. They consisted of eight women and one man with follow-up ranging from 0.8 to 13 years (Table 1). Time from tibial-nail placement until TKA ranged from 9 to 23 years; one patient without a record of nail placement was only able to give a rough estimate of implantation time (> 20 years). Staged removal of the intramedullary hardware occurred in six cases. Only one case involved complete removal of the nail and TKA performed concurrently. In two cases, the nail was partially removed and burred proximally to accommodate the tibial keel, but otherwise left in place. Cemented cruciate-retaining (CR) implants were used in all cases. Implant designs included four PFC Sigma (Depuy-Synthes, Warsaw, IN, USA), two Attune (Depuy-Synthes Warsaw, IN, USA), two Legion (Smith & Nephew, Memphis, TN, USA) and one iTotal (Conformis, Billerica, MA, USA). The cases which performed only a partial proximal-nail removal fitting the tibial keel posterior to the IMN included one PFC Sigma (Depuy-Synthes, Warsaw, IN, USA) and one Attune (Depuy-Synthes, Warsaw, IN, USA). All cases used an anterior midline incision incorporating the previous IMN incision. Case length was not recorded for two patients and, therefore, was not easily comparable between staged and non-staged cases; however, all cases performed in a non-staged fashion were > 130 min in length.

**Table 1:** Patient data, operative data, complications, outcomes. *IMN* intramedullary nail, *OKS* Oxford Knee Score

Patient no.	Age	Gender	BMI	IMN prior to TKA (years)	Nail Removed	Staged	Operative length (minutes)	Complication	Revision	Follow-up (years)	ROM	OKS
1	55	F	29	23	Yes	Yes	N/R		No	7.9	0–110	41
2	71	F	31	11	Yes	Yes	150		No	2.9	0–135	35
3	76	F	30	13	Yes	Yes	90		No	8.4	**N/R**	**N/R**
4	74	F	30	> 20	Yes	Yes	N/R		No	13	0–120	41
5	66	F	47	12	Yes	Yes	90		No	0.8	0–115	34
6	65	F	23	9	Yes	Yes	60		No	3.8	0–110	9
7	39	F	44	12	Partial	No	134	Arthrofibrosis, ankylosis, MUA × 2	No	7.2	30	16
8	53	F	34	22	Partial	No	140		No	3.3	5–115	23
9	69	M	31	20	Yes	No	134	Arthrofibrosis, MUA × 2	No	6	20–90	30

*BMI* Body Mass Index, *IMN* intramedullary nail, *MUA* manipulation under anesthesia, *N/R* not recorded, *OKS* Oxford Knee Score, *ROM* range of movement, *TKA* total knee arthroplasty

**Fig. 1:**
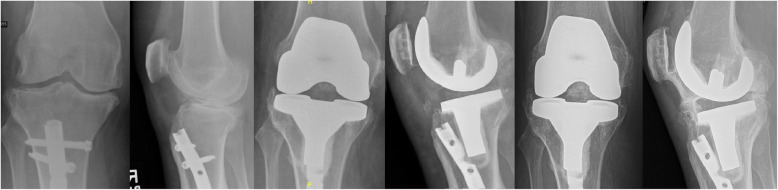
Preoperative, postoperative, and final follow-up radiographs of a patient who sustained joint ankylosis after conversion total knee arthroplasty (TKA) with partial intramedullary nail (IMN) removal

There were no complications related to wound healing or infection. No revisions were performed. No cases required stemmed or revision components. Two patients had arthrofibrotic knees, requiring multiple manipulations. Of note, both these patients underwent non-staged procedures. They both underwent manipulations with marginal benefit. One patient developed complete joint ankylosis (Fig. 1). Postoperative range of motion was highly variable as well as OKS ranging from 9 to 41. Patients who underwent a single surgery either with partial nail removal or complete nail removal reported poor OKS scores (16–30).

## Discussion

Despite the frequency of tibial-shaft fractures, few series have reported on TKA after tibial IMN fixation. Previous reports on TKA after open reduction and internal fixation (ORIF) have focused on tibial-plateau and distal femoral fractures [3, 10, 13, 14]. The few series which have included prior tibial fractures have either not identified whether intramedullary fixation was utilized or have not specifically evaluated these patients. Instead they were included as a small minority in a large heterogenous study group [3, 5]. We report the first series specifically evaluating patients who underwent conversion TKA after IMN. In the present study, the majority of cases involved complete nail removal prior to TKA performed in a staged fashion. This approach led to no complications with good functional outcomes.

Several technical adjustments may help accommodate placing a tibial implant without complete removal of intramedullary fixation. Utilizing implant systems with reduced tibial-keel length may decrease the likelihood of abutting existing hardware. Additionally, the use of cementless fixation requires less deep tibial preparation for placement of well-fixed implants. Lastly, the use of posterior-stabilized (PS) implants, as opposed to CR implants, requires less tibial slope and may reduce the likelihood of contact between the tibial keel and the IMN. With respect to these factors, we found no diversions from standard procedure with the included surgeons. As previously noted, in the present study, it appears that there was a preference to perform complete nail removal as opposed to altering these technical components during conversion arthroplasty. There was not a usage of reduced keel components. In fact, several of the implants have notoriously deep tibial keels. Additionally, all cases utilized cemented, CR implants (not PS).

Several authors have reported on challenges during conversion TKA after proximal tibial and distal femoral fractures. These include difficulties with exposure requiring tibial tubercle osteotomy, intraoperative disruption of the medial collateral ligament (MCL) and patellar tendons, increased use of stems and augments [8, 11–15]. Postoperative complications include increased postoperative stiffness, and wound complications [8, 12, 15, 16]. Weiss et al. reported a 3% rate of superficial infection and a 3% rate of deep infection when converting prior tibial-plateau fracture patients [13]. Saleh et al. reported an overall 20% rate of infection in these same type of conversion cases [12]. Scott et al. reported a trend toward increased wound complications where the previous incisions could not be easily incorporated into the approach for TKA [15]. Fortunately, this was not a challenge in our series as previous midline incisions were present in all patients. Regarding functional outcomes, these same studies had varied results. Several reported similar clinical scores comparing conversion patients to matched primary TKA patients while others reported decreased outcomes [12, 14–16].

Periarticular hardware, whether removed or retained, imparts increased perioperative complications during TKA. Kreitz et al. evaluated patients requiring periarticular hardware removal prior to TKA. Their cohort included cases of distal femoral and proximal tibial hardware from fractures and from corrective osteotomy. In this case group, they noted increased readmission and repeat procedures compared to matched primary TKA patients [3]. Interestingly, hardware retention does not appear to decrease this risk profile. Manrique et al. investigated patients with retained or partially retained periarticular hardware who underwent TKA and reported increased postoperative and mechanical complications and stiffness [9]. In our series, we identified two patients with partially retained hardware who both had poor outcome scores by OKS. One patient eventually developed complete joint ankylosis (Fig. 1).

The value of staged vs non-staged hardware removal is still undetermined. Bergen et al. looked at conversion TKA cases comparing staged and non-staged hardware removal. They found no differences in resource utilization or postoperative mechanical or wound complications comparing these two study groups. However, they reported on a widely heterogenous group of cases and hardware types from anterior cruciate ligament (ACL) screws to distal femoral plates [10]. Selection bias surely affected this comparison as more complex cases likely underwent staged hardware removal. Lizaur-Utrilla et al. performed staged hardware removal in cases of prior tibial-plateau fracture and noted decreased wound complications when comparing their data to previous series performed in a non-staged fashion [12–14]. We present six cases of staged IMN removal prior to TKA. Unfortunately, operative data were lacking in two of the cases. Without nail removal, these cases were likely shorter in duration than cases involving concomitant removal or partial removal. Cases involving staged nail removal had excellent postoperative range of movement (ROM). Except for one patient, these patients had excellent OKS scores. Two of the three cases not utilizing staged hardware removal developed stiffness, with one patient developing ankylosis. These patients reported poor OKS scores at 3.3–7.2 years after surgery. We saw no increased infectious complications in patients who underwent two surgeries. Despite hypothesizing that staged tibial-nail removal would result in increased complications this was not reported. In our opinion, it is reasonable to perform staged hardware removal with an interval period to maintain ROM and limit potential for postoperative stiffness.

Limitations of this series include the small patient number, and retrospective nature of the analysis. Additionally, several of the cases provide minimal preoperative information and operative details are lacking in two cases. For instance, information about preoperative functional scores and range of motion were lacking for several patients. Postoperatively, one patient could not be contacted for functional assessment. Future studies should include multicenter data to capture more study subjects. Despite shortcomings, this represents the first series presenting cases of TKA after tibial IMN fixation and is instructive for surgeons who will likely encounter this clinical scenario more frequently as the rate of knee replacement continues to grow.

## Conclusions

Patients with prior tibial IMN fixation present unique challenges when performing TKA. While underrepresented in the literature currently, this rare scenario will likely increase as the need for knee arthroplasty increases. Conversion TKA after tibial IMN fixation imparts similar intraoperative and postoperative challenges to more extensively studied conversion TKA scenarios. There are multiple technical factors which may assist in placement of tibial components without complete nail removal. However, complete nail removal prior to TKA simplifies the technical portion of the reconstruction and was the preferred technique in the present study. Both staged and non-staged hardware removal appear to be adequate techniques but more power is required to detect differences in outcomes.

## Data Availability

The datasets during and/or analyzed during the current study are available from the corresponding author on reasonable request.
